# STAT2-Dependent Immune Responses Ensure Host Survival despite the Presence of a Potent Viral Antagonist

**DOI:** 10.1128/JVI.00296-18

**Published:** 2018-06-29

**Authors:** Vu Thuy Khanh Le-Trilling, Kerstin Wohlgemuth, Meike U. Rückborn, Andreja Jagnjic, Fabienne Maaßen, Lejla Timmer, Benjamin Katschinski, Mirko Trilling

**Affiliations:** a Institute for Virology, University Hospital Essen, University Duisburg-Essen, Essen, Germany; University of Southern California

**Keywords:** STAT transcription factors, cytomegalovirus, immune antagonists, *in vivo*, interferons

## Abstract

A pathogen encounter induces interferons, which signal via Janus kinases and STAT transcription factors to establish an antiviral state. However, the host and pathogens are situated in a continuous arms race which shapes host evolution toward optimized immune responses and the pathogens toward enhanced immune-evasive properties. Mouse cytomegalovirus (MCMV) counteracts interferon responses by pM27-mediated degradation of STAT2, which directly affects the signaling of type I as well as type III interferons. Using MCMV mutants lacking *M27* and mice lacking STAT2, we studied the opposing relationship between antiviral activities and viral antagonism in a natural host-pathogen pair *in vitro* and *in vivo*. In contrast to wild-type (wt) MCMV, ΔM27 mutant MCMV was efficiently cleared from all organs within a few days in BALB/c, C57BL/6, and 129 mice, highlighting the general importance of STAT2 antagonism for MCMV replication. Despite this effective and relevant STAT2 antagonism, wt and STAT2-deficient mice exhibited fundamentally different susceptibilities to MCMV infections. MCMV replication was increased in all assessed organs (e.g., liver, spleen, lungs, and salivary glands) of STAT2-deficient mice, resulting in mortality during the first week after infection. Taken together, the results of our study reveal the importance of cytomegaloviral interferon antagonism for viral replication as well as a pivotal role of the remaining STAT2 activity for host survival. This mutual influence establishes a stable evolutionary standoff situation with fatal consequences when the equilibrium is disturbed.

**IMPORTANCE** The host limits viral replication by the use of interferons (IFNs), which signal via STAT proteins. Several viruses evolved antagonists targeting STATs to antagonize IFNs (e.g., cytomegaloviruses, Zika virus, dengue virus, and several paramyxoviruses). We analyzed infections caused by MCMV expressing or lacking the STAT2 antagonist pM27 in STAT2-deficient and control mice to evaluate its importance for the host and the virus *in vitro* and *in vivo*. The inability to counteract STAT2 directly translates into exaggerated IFN susceptibility *in vitro* and pronounced attenuation *in vivo*. Thus, the antiviral activity mediated by IFNs via STAT2-dependent signaling drove the development of a potent MCMV-encoded STAT2 antagonist which became indispensable for efficient virus replication and spread to organs required for dissemination. Despite this clear impact of viral STAT2 antagonism, the host critically required the remaining STAT2 activity to prevent overt disease and mortality upon MCMV infection. Our findings highlight a remarkably delicate balance between host and virus.

## INTRODUCTION

Human cytomegalovirus (HCMV), a prototypic betaherpesvirus, is a ubiquitously distributed virus with clinical importance. After primary infection, the virus is usually controlled but not eliminated by the host immune system. HCMV establishes lifelong latent infections from which the virus can reactivate and cause recurrent disease. In the absence of adequate immunity, the otherwise mostly subclinical infection can cause profound morbidity and mortality. As a result of millions of years of coevolution, HCMV replication is highly adapted to the human host and became extremely species specific. *In natura*, viral replication is restricted to Homo sapiens. Therefore, meaningful experiments with HCMV in small animals are impossible, maybe with the notable exception of aspects assessable in humanized mice (1, 2). To allow studies on cytomegalovirus (CMV) pathogenesis *in vivo*, related CMVs naturally infecting animals, like rhesus monkey, guinea pig, rat, and mouse, are applied as model systems. Mouse cytomegalovirus (MCMV; *Murid herpesvirus 1*; taxonomic identifier, 10366) is one of the most well-established models for HCMV and is also used to study general phenomena in basic immunological and virological research. Since Mus musculus is the natural host of MCMV, as evident from the fact that free-ranging mouse populations show very high MCMV seroprevalences (3), MCMV infections constitute one of the few models in which the history of reciprocal adaptations is taken into account. This is important for the understanding of immunity and viral immune evasion since reciprocal adaptation shapes the protein functions of the host and virus (4).

Cytomegaloviruses express numerous immune-evasive gene products targeting most aspects of intrinsic, innate, and adaptive immunity (e.g., see references 5 and 6). The focus of this study was the antithetic relationship between the interferon (IFN) system and virus-encoded IFN antagonists. Reports dating back to 1967 had already documented a remarkable IFN resistance of HCMV (7). This insusceptibility is achieved by HCMV-encoded inhibitors of the IFN system and is conserved in related CMVs, like MCMV. Beside its ability to inhibit the induction of IFNs (8, 9), MCMV encodes at least two gene products counteracting different aspects of Jak-STAT downstream signaling (10). One of these inhibitors is pM27, which specifically antagonizes STAT2-dependent Jak-STAT signaling, enabling virus replication in the presence of type I and type II IFNs (11). The protein pM27 interacts with STAT2 and DDB1/Cullin4A/B/RocA ubiquitin ligase complexes, leading to ubiquitination and the subsequent proteasomal degradation of STAT2 (12). Since type III IFNs also signal via STAT2-containing complexes (13), viral degradation of STAT2 should also influence type III IFNs, but such an effect has not been described so far. HCMV also reduces STAT2 protein amounts (14, 15) by a mechanism which is sensitive to inhibitors of the proteasome (14). Nevertheless, pUL27, the homolog of pM27, is dispensable for HCMV-mediated STAT2 degradation (14). To our knowledge, the responsible HCMV gene product(s) mediating STAT2 degradation has not been described so far. The importance of Cullin activity for pM27-dependent STAT2 degradation is evident from its MLN4924 sensitivity (16). The drug MLN4924 inhibits the Nedd8-activating enzyme, which is essential for Cullin/Roc ubiquitin ligase (CRL) activity (17). A global proteome profiling approach revealed that MLN4924 treatment also restores STAT2 amounts in HCMV-infected cells (16), indicating that CRLs are, as in the case of MCMV, involved in the expression and/or function of the HCMV-encoded STAT2 antagonist(s). Thus, MCMV constitutes a suitable model for HCMV since both viruses induce proteasomal STAT2 degradation involving CRLs. The importance of pM27 for MCMV replication *in vivo* first became evident when Liu and coworkers described the attenuation phenotype of an MCMV mutant harboring a transposon insertion in the *M27* gene (18). Consistently, we characterized the function of pM27 and observed that a targeted deletion of *M27* strongly attenuates MCMV *in vivo* replication, which is recovered to a certain extent in mice lacking either the receptor IFNAR1 or the receptor IFNGR1 (11).

Several viruses encode efficient antagonists targeting the IFN-Jak-STAT system and STAT2 in particular (e.g., dengue virus [19], Zika virus [20], and several parainfluenza viruses [21–23]). The relevance of STAT2 is further underlined by the finding that humans suffering from loss-of-function mutations in *STAT2* exhibit a pronounced susceptibility toward viral infections, including those with attenuated vaccine strains (24–27). This raises the question concerning the role of STAT2 for the host and the relevance of the antagonism for virus replication. In particular, we asked if antagonists achieve complete elimination of the protective effects induced via STAT2 or if STAT2 mounts protective effects despite the presence of viral evasion strategies.

Our comprehensive analysis of MCMV replication in the absence of either the viral pM27-mediated STAT2 antagonism or the STAT2-coding capacity of the host revealed an evolutionary standoff situation and highlights a remarkably delicate balance between host and virus in which no side can afford to cede control over STAT2. Even in the presence of viral countermeasures, the remaining antiviral activity induced by STAT2 defines host survival.

## RESULTS

### ΔM27-MCMV is highly attenuated *in vivo* irrespective of viral MCK-2 functionality.

**FIG 1:**
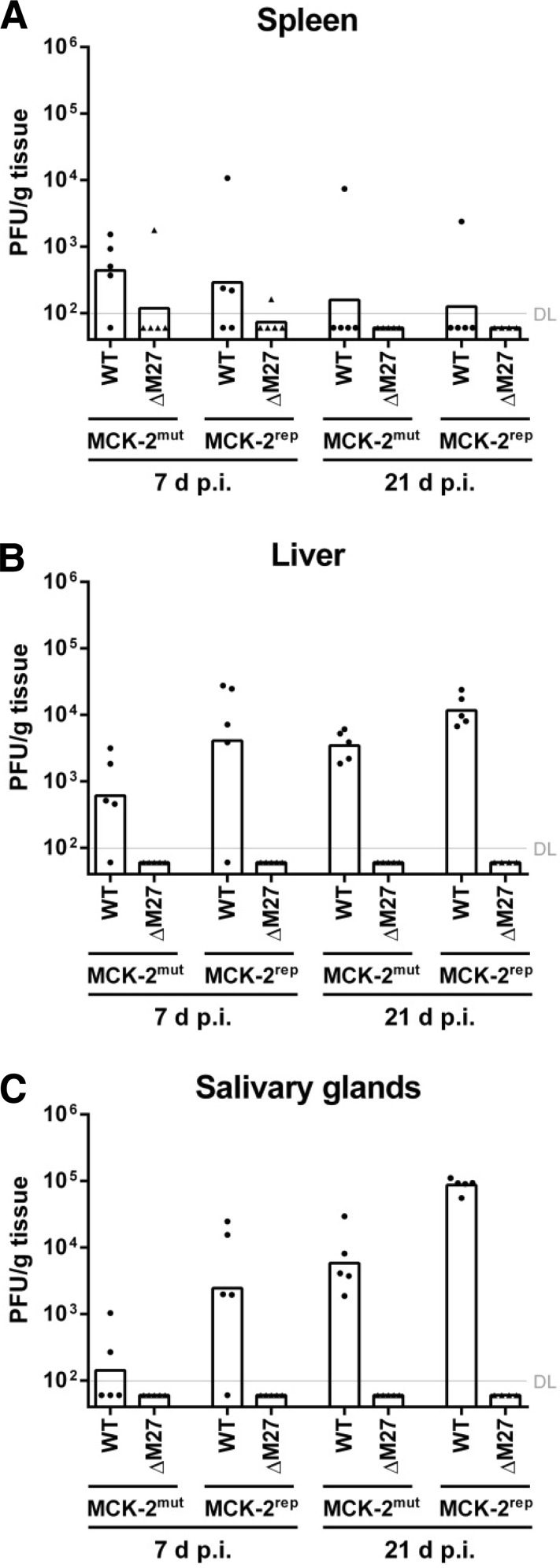
ΔM27-MCMV is highly attenuated *in vivo* irrespective of viral MCK-2 functionality. C57BL/6 mice were infected i.p. with 2 × 10^5^ PFU of BAC-derived MCMV with mutated and repaired MCK-2. At 7 and 21 days postinfection (d p.i.), the indicated organs were harvested and frozen. The virus titers were determined from organ homogenates by plaque titration. The titrations were done in quadruplicate. Bars depict the geometric mean; dots and triangles show the titers for individual mice (*n* = 4 to 5). DL, detection limit. (A) Virus titer of spleens. (B) Virus titer of livers. (C) Virus titer of salivary glands.

Previous work relied on either a mutant harboring a transposon insertion in the *M27* coding sequence (CDS) (18) or a targeted MCMV mutant generated on the background of the pSM3fr bacterial artificial chromosome (BAC) (11). The latter contains a loss-of-function mutation in the *MCK-2* gene which impairs viral replication in the salivary glands (28). MCK-2 binds the glycoprotein gH and is required for the recruitment (29, 30) and infection (31) of macrophages. Thus, previous work could have been confounded by unspecific and/or distal effects associated with the transposon insertion or the fact that a double mutant (*M27* and *MCK-2*) was studied. To exclude this possibility, a targeted ΔM27 mutant MCMV isolate (ΔM27-MCMV) was generated based on an *MCK-2*-repaired BAC. Due to previous publications showing a strong attenuation (11), we compared the *in vivo* replication of wild-type MCMV (wt-MCMV) and ΔM27-MCMV on an *MCK-2* mutated (MCK-2^mut^) and -repaired (MCK-2^rep^) background (Fig. 1A to C). Consistent with results published by Jordan and coworkers (28), the *MCK-2* mutation affected wt-MCMV replication, especially in the salivary glands (Fig. 1C). Irrespective of the MCK-2 functionality, ΔM27-MCMV was severely attenuated at 7 and 21 days postinfection (p.i.) in the spleen, liver, and salivary glands of infected C57BL/6 mice. In most animals and organs, no ΔM27-MCMV replication could be detected (Fig. 1A to C). This indicates that a deletion of the *M27* CDS attenuates primary MCMV replication in C57BL/6 mice, irrespective of the absence or presence of additional *MCK-2* mutations. Nevertheless, we used MCMVs with a repaired *MCK-2* gene for all further experiments described here.

### ΔM27-MCMV is highly attenuated *in vivo*, irrespective of the presence or absence of Ly49H.

**FIG 2:**
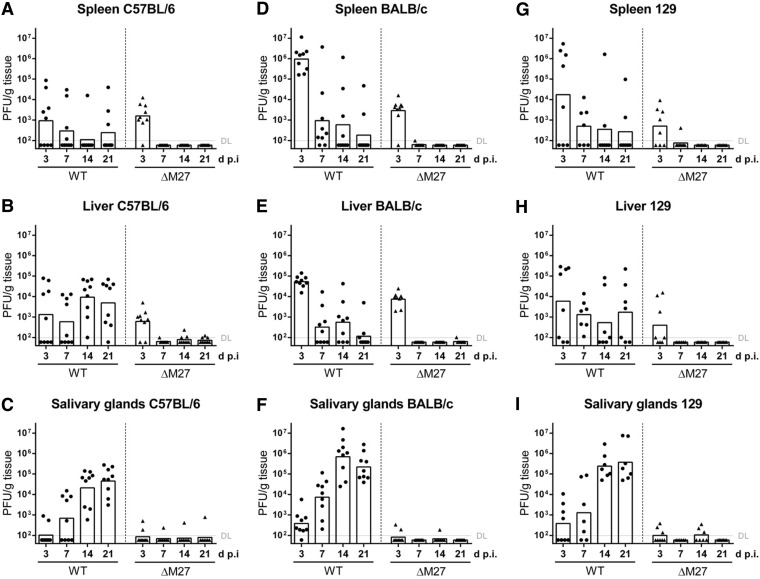
ΔM27-MCMV is highly attenuated *in vivo* irrespective of the presence or absence of Ly49H. C57BL/6, BALB/c, and 129 mice were infected i.p. with 2 × 10^5^ PFU of wt-MCMV and ΔM27-MCMV. At 3, 7, 14, and 21 days p.i., the indicated organs were harvested and frozen. The virus titers were determined from organ homogenates by plaque titration. The titrations were done in quadruplicate. Pooled data from two independent experiments are shown. Bars depict the geometric mean; dots and triangles show the titers for individual mice (*n* = 7 to 9). DL, detection limit. (A) Virus titer of C57BL/6 mouse spleens. (B) Virus titer of C57BL/6 mouse livers. (C) Virus titer of C57BL/6 mouse salivary glands. (D) Virus titer of BALB/c mouse spleens. (E) Virus titer of BALB/c mouse livers. (F) Virus titer of BALB/c mouse salivary glands. (G) Virus titer of 129 mouse spleens. (H) Virus titer of 129 mouse livers. (I) Virus titer of 129 mouse salivary glands.

Mouse strains differ significantly concerning their MCMV susceptibility (32, 33). One genetic locus determining this predisposition (named *cmv1* [34]) codes for Ly49H (35–37). In susceptible mice, the MCMV-encoded major histocompatibility complex-like molecule pm157 interacts with Ly49H, resulting in pronounced NK cell activation (38, 39). NK cells produce large quantities of gamma interferon (IFN-γ), e.g., upon pm157-dependent Ly49H activation (40). Additionally, NK cells indirectly affect IFN-γ secretion by T lymphocytes, e.g., by acting as “rheostats, or master regulators, of antiviral T-cell responses” (41). Since ΔM27-MCMV exhibits a pronounced *in vitro* susceptibility toward IFN-γ (11, 12), we compared Ly49H-positive (C57BL/6) and Ly49H-negative (BALB/c) mouse strains concerning ΔM27-MCMV susceptibility. Consistent with previous publications (e.g., see reference 32), we observed that early (at 3 days p.i.) wt-MCMV replication in spleen and liver is largely impaired in Ly49H-positive C57BL/6 mice (Fig. 2A and B for C57BL/6 mice and 2D and E for BALB/c mice). Interestingly, at later time points, considerable MCMV replication was observed not only in Ly49H-negative BALB/c mice (Fig. 2F) but also in the liver and salivary glands of Ly49H-positive C57BL/6 mice (Fig. 2B and C). At 3 days p.i., ΔM27-MCMV replication was detected to a certain extent in spleen and liver of C57BL/6 and BALB/c mice (Fig. 2A and B for C57BL/6 mice and 2D and E for BALB/c mice). At later times postinfection (7 to 21 days p.i.) and in the salivary glands, only occasional events of low-level ΔM27-MCMV replication were found in individual mice (Fig. 2A to F). To rule out the possibility of wt-MCMV contaminations, we reisolated ΔM27-MCMV at 3 days p.i. from the spleen and confirmed the absence of *M27* by PCR (data not shown). In addition to Ly49H, pm157 also interacts with the inhibitory NK receptor Ly49I, which is expressed by mice of the Ly49H-negative 129 strain (38). To test the relevance of Ly49I and to enable experiments using genetically modified mice generated on this background, mice of the 129 strain were infected with wt-MCMV or ΔM27-MCMV and organ titers were determined. Similar to the results obtained for C57BL/6 and BALB/c mice, we observed a severe attenuation of ΔM27-MCMV (Fig. 2G to I). When the results are taken together, ΔM27-MCMV shows a transient episode of low-level replication in spleen and liver early after infection and exhibits a very pronounced attenuation in an Ly49H/I-independent manner. These data indicate that STAT2 antagonism by pM27 is a prerequisite for efficient *in vivo* replication and to reach organs required for dissemination (e.g., the salivary glands). This phenomenon dominates over mouse strain-specific phenotypes of resistance and susceptibility.

### The *in vitro* IFN susceptibility of ΔM27-MCMV is fully reversed by pharmacologic inhibition of Janus kinase activity and in the absence of STAT2.

**FIG 3:**
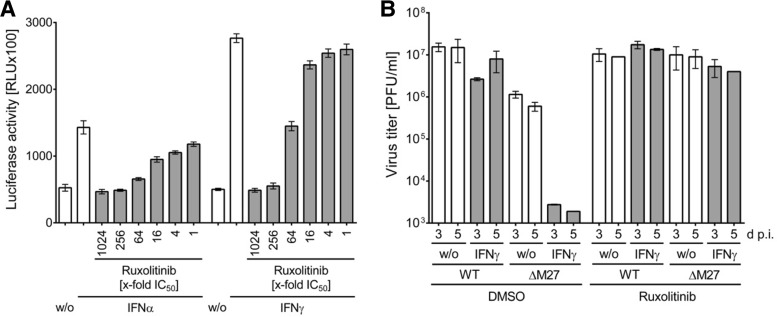
Pharmacologic inhibition of Janus kinase activity completely reverts the *in vitro* IFN-γ susceptibility of ΔM27-MCMV. (A) 3T3:ISREluc reporter cells (11) were left untreated or were treated with 250 U/ml of IFN-α and IFN-γ in the absence or presence of decreasing concentrations of ruxolitinib. After 6 h of treatment, cells were lysed and luciferase activity was quantified. Measurements were performed in triplicate. Mean values ± the standard deviations (SD) are shown. RLU, relative light units. (B) Immortalized mouse fibroblasts were left untreated or were incubated for 48 h with IFN-γ (250 U/ml) before infections using wt-MCMV and ΔM27-MCMV were done (multiplicity of infection, 0.1). The infection was done in the absence or presence of 4 μM ruxolitinib (ca. 1,200-fold the 50% inhibitory concentration [IC_50_]). At 3 and 5 days p.i., virus titers were quantified. Each titration was done in triplicate. Mean values ± SD are shown. w/o, no treatment; DMSO, dimethyl sulfoxide.

The replication of ΔM27-MCMV *in vitro* is highly susceptible toward type I and type II IFNs. Especially, IFN-γ treatment prevents ΔM27-MCMV replication almost entirely (11, 12). However, several viral proteins harbor more than one biological function. Additionally, IFNs might overproportionally affect mutants with impaired overall fitness, even if the mutation does not affect genes directly involved in IFN antagonism. To test if *M27* deficiency can be reverted by blocking IFN signaling, ruxolitinib was applied to inhibit Janus kinase activity and downstream STAT signaling. Ruxolitinib efficiently and dose dependently blocked IFN-α- and IFN-γ-induced gene expression in an IFN-stimulated response element (ISRE) reporter cell line (Fig. 3A). Ruxolitinib addition prevented the slight replication impairment of ΔM27-MCMV in untreated cells (Fig. 3B), suggesting that endogenously produced IFNs cause the reduced replication. Coadministration of ruxolitinib together with IFN-γ at 48 h prior to infection (data not shown) as well as IFN-γ preincubation followed by ruxolitinib addition at the time point of infection, thereby phenocopying the presence of the Jak-STAT inhibitor pM27, completely reverted the pronounced antiviral activity of IFN-γ and normalized ΔM27-MCMV replication to wt-MCMV levels (Fig. 3B).

**FIG 4:**
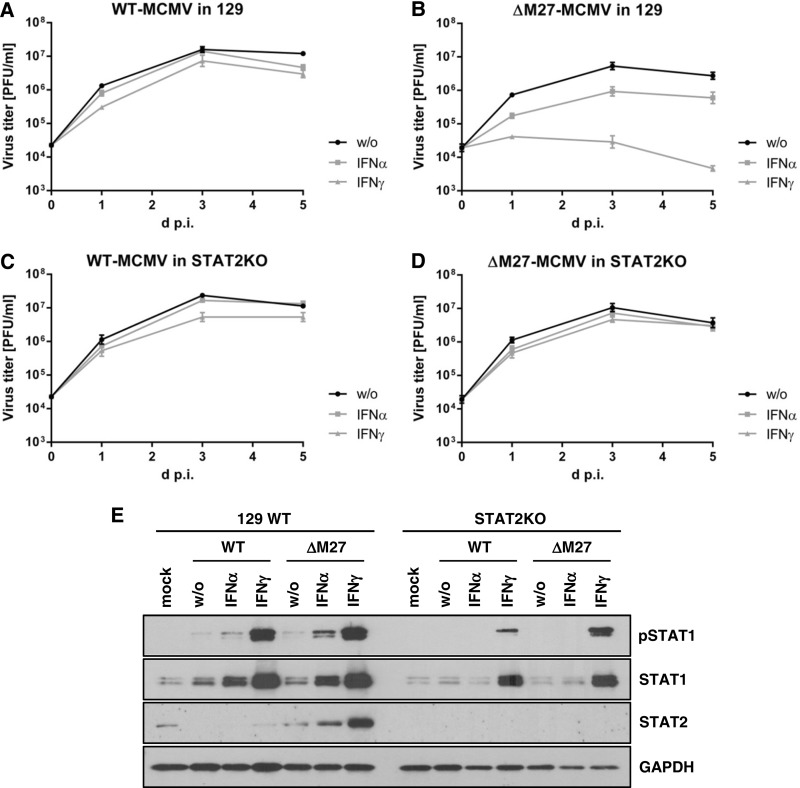
The *in vitro* IFN susceptibility of ΔM27-MCMV is fully reversed in the absence of STAT2. Primary mouse newborn cells (MNC) isolated from 129 and STAT2-knockout mice were left untreated or were preincubated for 48 h with 250 U/ml IFN before they were infected with wt-MCMV and ΔM27-MCMV. Virus titers were quantified at the indicated time points postinfection. Each titration was done in triplicate. Mean values ± SD are shown. (A) Titer of wt-MCMV in 129 cells. (B) Titer of ΔM27-MCMV in 129 cells. (C) Titer of wt-MCMV in STAT2-knockout (STAT2KO) cells. (D) Titer of ΔM27-MCMV in STAT2-knockout cells. (E) Protein lysates were generated in parallel to the replication analysis and under the same experimental conditions. Immunoblot analysis of the indicated proteins was performed. pSTAT1, phospho-STAT1.

To test if the STAT2-degrading function is the cause of the observed ΔM27-MCMV phenotype, the IFN susceptibility of wt-MCMV and ΔM27-MCMV was assessed in primary mouse newborn cells (MNC) from STAT2-deficient and wild-type mice. The replication of wt-MCMV was almost completely resistant to pretreatment with IFN-α and IFN-γ, whereas ΔM27-MCMV replication was highly susceptible toward IFNs (Fig. 4A and B). In IFN-γ-treated cells, ΔM27-MCMV replication was virtually prevented (Fig. 4B). In clear contrast, STAT2-deficient cells allowed replication of ΔM27-MCMV comparable to that of wt-MCMV, irrespective of IFN treatment (Fig. 4C and D). Since it was reported that the absence of STAT2 influences STAT1 protein levels in certain cell types (e.g., in primary embryonic fibroblasts, but not in macrophages) (42), presumably due to impaired constitutive IFN signaling (43, 44), we tested STAT1 amounts by immunoblotting in parallel to the replication analysis under the same experimental conditions. As shown in Fig. 4E, STAT2-deficient MNCs had STAT1 protein levels comparable to those of wild-type MNCs (see lanes mock). Infection with wt-MCMV reduced STAT2 levels in wild-type cells, whereas ΔM27-MCMV lost the ability to degrade STAT2. As expected, IFN-α responsiveness was observed only for wild-type cells and not for STAT2-deficient cells. IFN-γ responsiveness (as measured by the phosphorylation of STAT1 and an increase in STAT1 expression) was preserved in STAT2-deficient MNCs, albeit in a moderately reduced manner (Fig. 4E). Taken together, these findings indicate that the entire IFN susceptibility of ΔM27-MCMV *in vitro* is STAT2 dependent and that the biologically relevant function of pM27 suggested by phenotypic analyses in cell culture is the antagonism of Jak-STAT signaling. Additionally, these data further support previous reports which described a contribution of STAT2 to antiviral IFN-γ responses (11, 12, 45, 46).

### The protein pM27 inhibits IFN-λ signaling.

**FIG 5:**
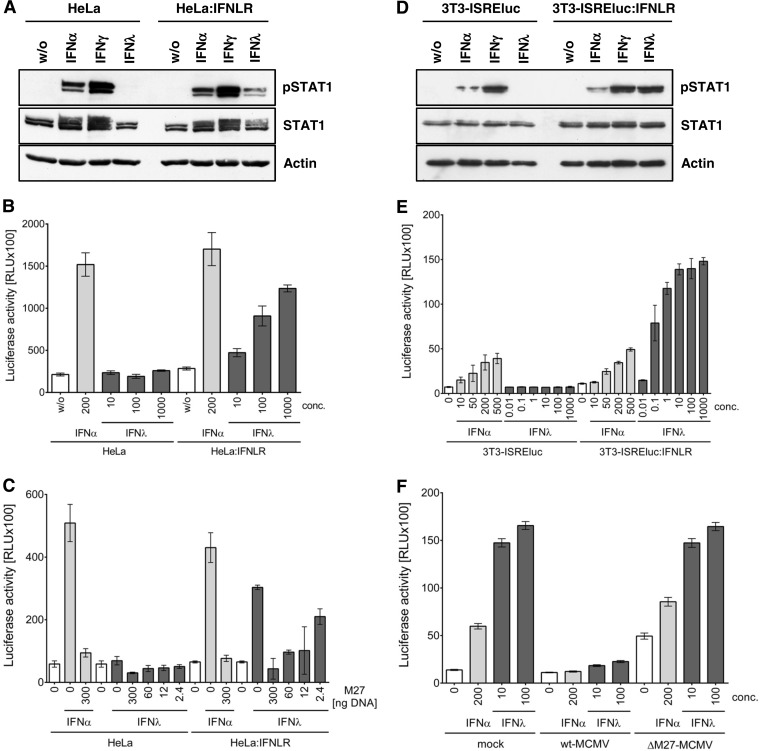
pM27 inhibits IFN-λ signaling. For the analysis of IFN-λ signaling, HeLa:IFNLR and 3T3-ISREluc:IFNLR cells stably expressing IFNLR1 were generated. (A) Parental control HeLa and HeLa:IFNLR cells were treated with the indicated IFN type for 15 min. Cells were lysed, and immunoblot analysis of the indicated proteins was performed. (B) HeLa and HeLa:IFNLR cells were transfected with an ISRE-luciferase reporter plasmid before they were treated with the indicated doses of IFN-α and IFN-λ (in units per milliliter and nanograms per milliliter, respectively). After 5 h of treatment, cells were lysed and luciferase activity was determined. Each condition was measured in triplicate. Mean values ± SD are shown. (C) As in panel B, but the cells were additionally cotransfected with decreasing amounts of an *M27* expression plasmid (12) before they were treated with IFN-α (200 U/ml) or IFN-λ (1,000 ng/ml). (D) Parental 3T3-ISREluc and 3T3-ISREluc:IFNLR cells were treated with the indicated IFN type for 15 min. Cells were lysed, and immunoblot analysis of the indicated proteins was performed. (E) 3T3-ISREluc and 3T3-ISREluc:IFNLR cells were treated with the indicated doses of IFN-α and IFN-λ (in units per milliliter and nanograms per milliliter, respectively). After 5 h of treatment, cells were lysed and luciferase activity was determined. Each condition was measured in quadruplicate. Mean values ± SD are shown. (F) 3T3-ISREluc:IFNLR cells were infected with wt-MCMV and ΔM27-MCMV (multiplicity of infection, 3). At 24 h p.i., cells were treated with the indicated IFN. After 5 h of treatment, cells were lysed and luciferase activity was determined. Each condition was measured in quadruplicate. Mean values ± SD are shown.

It is well-known that STAT2 is part of the type I IFN signal transduction pathway, but STAT2 is also activated by type III IFNs (13). Unlike type I IFNs, whose receptor is ubiquitously expressed, type III IFNs (IFN-λ) preferentially act at epithelial surfaces (47, 48), as the expression of the IFN-λ receptor is restricted to certain cell types, like epithelial cells (47). Since pM27 targets STAT2, we hypothesized that the pM27 function might also affect IFN-λ responses. To address this, we generated a HeLa cell line (HeLa:IFNLR) which stably expresses the human IFNLR1. We chose HeLa cells since they are readily transfectable and pM27 is able to degrade mouse as well as human STAT2 (12). HeLa:IFNLR cells respond to IFN-λ, as is apparent by detection of phospho-STAT1 and in ISRE reporter assays (Fig. 5A and B). When we cotransfected HeLa:IFNLR cells with a pISRE reporter plasmid together with decreasing amounts of a pM27 expression construct, a dose-dependent inhibition of IFN-λ-induced ISRE promoter activity was observed (Fig. 5C). This result prompted us to determine the importance of pM27 for inhibition of IFN-λ signaling in MCMV-infected cells. To this end, we stably expressed mouse IFNLR1 in a previously described (11) NIH 3T3-based ISRE luciferase reporter cell line (3T3-ISREluc:IFNLR). These 3T3-ISREluc:IFNLR cells were responsive to IFN-λ treatment (Fig. 5D and E) and permissive to MCMV infection (data not shown). Using the 3T3-ISREluc:IFNLR cells, we observed that wt-MCMV blocked IFN-λ-induced gene expression, whereas ΔM27-MCMV lost this inhibitory capacity (Fig. 5F). Our data show that pM27 antagonizes IFN-λ signaling and highlight the essential role of STAT2 for type III IFN responses.

### STAT2-deficient mice are highly susceptible to MCMV infection.

**FIG 6:**
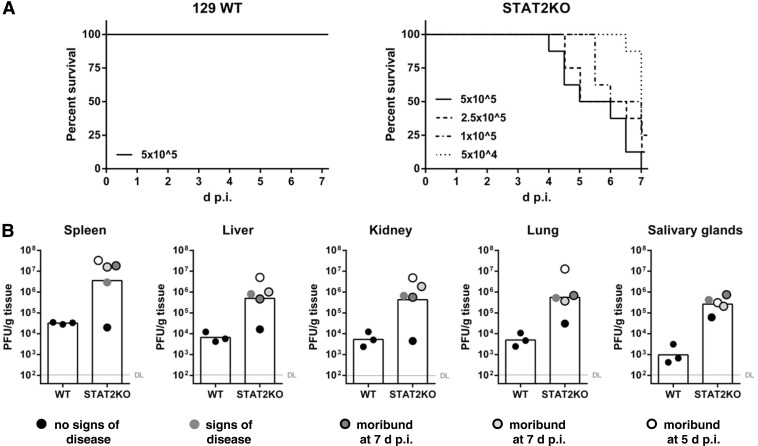
STAT2-deficient mice are highly susceptible to MCMV infection. (A) 129 and STAT2-knockout (STAT2KO) mice were infected i.p. with the indicated doses of wt-MCMV. Infected animals were inspected twice a day. The number of moribund and dead mice was monitored to calculate the survival rates. The percentages of surviving animals (not moribund or dead) are depicted. Pooled data from two independent experiments are shown (*n* = 7 for 129 mice and *n* = 8 for STAT2-knockout mice). (B) High MCMV replication correlates with disease severity in STAT2-deficient mice. 129 and STAT2-knockout mice were infected i.p. with 2 × 10^5^ PFU of wt-MCMV. At 5 days p.i. (1 STAT2-knockout mouse) and 7 days p.i. (all other mice), the indicated organs were harvested and frozen. The virus titers were determined from organ homogenates by plaque titration. The titrations were done in quadruplicate. Bars depict the geometric mean; dots show the titers for individual mice (*n* = 5). The virus titers in the indicated organs are shown. DL, detection limit. Additionally, the infected mice were assigned according to the severity of disease into the following groups: no signs of disease (black circles), signs of disease (gray circle), moribund at 7 days p.i. (black-rimmed gray circles), and moribund at 5 days p.i. (black-rimmed white circle).

Having unequivocally documented that MCMV efficiently counteracts STAT2-dependent innate immunity and that this ability is essential for replication in IFN-experienced cells *in vitro* as well as for efficient viral replication and propagation *in vivo*, we raised questions concerning the selective advantage for the host to mount and maintain functional STAT2 immune responses. Therefore, experiments with STAT2-deficient and 129 wild-type control mice were performed. When STAT2-deficient mice were infected with wt-MCMV—from which we know that it efficiently counteracts IFN induction (8) as well as Jak-STAT signaling, the latter by at least two independent gene products (10), one of which targets STAT2 (10–12)—we expected an increased susceptibility, but we were surprised by the severity of the phenotype: within 1 week, STAT2-deficient mice infected with the standard MCMV dose (2 × 10^5^ PFU per mouse) showed obvious signs of disease, and more than half of the mice were moribund or had died (data not shown). None of the wild-type animals showed similar disease or succumbed to infection (data not shown and Fig. 6A). When the virus inoculum was titrated using doses ranging from 5 × 10^4^ to 5 × 10^5^ PFU per mouse, most infected STAT2-deficient mice showed obvious signs of disease and became moribund. The percentage of surviving animals inversely correlated with the dose of the virus inoculum (Fig. 6A). Since STAT2-mediated IFN signaling is impaired by MCMV very efficiently, this severe and fast progression of disease of MCMV-infected STAT2-deficient mice demonstrated that the remaining STAT2 activity is essential to prevent overt disease upon MCMV infection.

### High MCMV replication correlates with disease severity in STAT2-deficient mice.

The increase in morbidity and mortality upon MCMV infection of STAT2-deficient mice raised the question concerning the MCMV titers in these mice. Due to the lethality of the MCMV infection in STAT2-deficient mice, only very early time points could be analyzed. STAT2-deficient and 129 control mice were infected and monitored regularly to assign the severity of disease. One infected STAT2-deficient mouse was moribund at 5 days p.i. and had to be sacrificed earlier than the other animals (Fig. 6B). The remaining mice were sacrificed at 7 days p.i., when most of the STAT2-deficient mice were moribund. The spleen, liver, kidney, lung, and salivary glands of all animals were harvested to determine the viral load. The STAT2-deficient mice showed a generalized infection, with the MCMV titers in all tested organs being higher than those in the tested organs of the control mice and with the titers being several orders of magnitude higher than those in the control mice (Fig. 6B). High viral titers were also observed in kidney and lungs (Fig. 6B), where MCMV replication is normally low (data not shown). We also found that the MCMV titers correlated with the disease severity: only the one STAT2-deficient mouse with lower MCMV replication showed no signs of disease (Fig. 6B), suggesting that the viral replication itself causes the pathology.

### The *in vivo* attenuation of ΔM27-MCMV is largely restored in STAT2-deficient mice.

**FIG 7:**
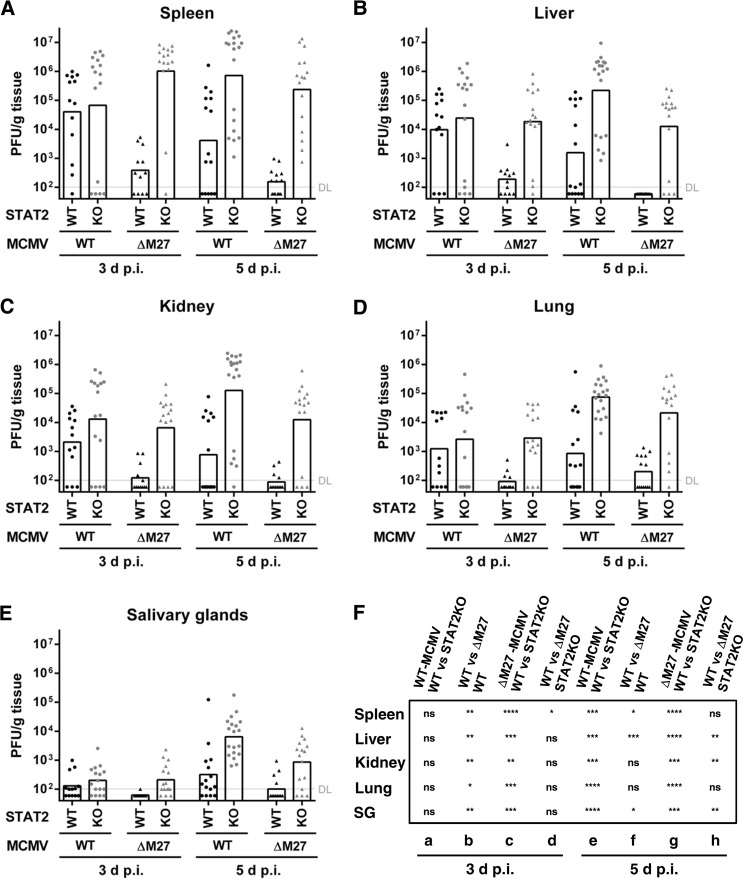
The *in vivo* attenuation of ΔM27-MCMV is largely restored in STAT2-deficient mice. 129 and STAT2-knockout mice were infected i.p. with 2 × 10^5^ PFU of wt-MCMV and ΔM27-MCMV. At 3 and 5 days p.i., the indicated organs were harvested and frozen. The virus titers were determined from organ homogenates by plaque titration. The titrations were done in quadruplicate. Pooled data from three independent experiments are shown. Bars depict the geometric mean; dots and triangles show the titers for individual mice (*n* = 12 to 15 for 129 mice and *n* = 16 to 19 for STAT2-knockout mice). DL, detection limit. (A) Virus titer of spleens. (B) Virus titer of livers. (C) Virus titer of kidneys. (D) Virus titer of lungs. (E) Virus titer of salivary glands. (F) The significance of differences among groups was analyzed by the Mann-Whitney test. ns, not significant; *, *P* < 0.05; **, *P* < 0.01; ***, *P* < 0.001; ****, *P* < 0.0001. SG, salivary glands.

Our previous analysis (Fig. 3 and 4) implied no biologically relevant pM27-dependent effects beyond antagonism of STAT2 and Jak-STAT signaling *in vitro*. However, the impact of potential additional functions of pM27 could be restricted to the *in vivo* situation in which ΔM27-MCMV is severely impaired. To analyze this, control mice of the 129 strain and STAT2-deficient mice were infected with wt-MCMV and ΔM27-MCMV. At 3 and 5 days p.i., the replication in the spleen, liver, kidney, lungs, and salivary glands was assessed. As expected, ΔM27-MCMV replication was attenuated in 129 mice compared to wt-MCMV replication (Fig. 7A to E). Consistent with the above-mentioned *in vitro* findings, the replication of ΔM27-MCMV was largely restored in STAT2-deficient mice (Fig. 7A to E), indicating that the strong *in vivo* attenuation is indeed a direct consequence of the lack of an ability to counteract the STAT2-dependent induction of an antiviral state. Interestingly, the difference between viral replication in wild-type and STAT2-deficient mice was more pronounced at 5 days p.i. than at 3 days p.i. (especially in the case of wt-MCMV; see also Fig. 7F, column a versus column e), indicating that the control of the virus after the first wave of replication critically depends on STAT2. Interestingly, a small but significant difference between wt-MCMV and ΔM27-MCMV titers in STAT2-deficient mice was found in certain organs (Fig. 7F). This finding indicates that pM27 possesses at least one additional function beyond STAT2 antagonism.

**FIG 8:**
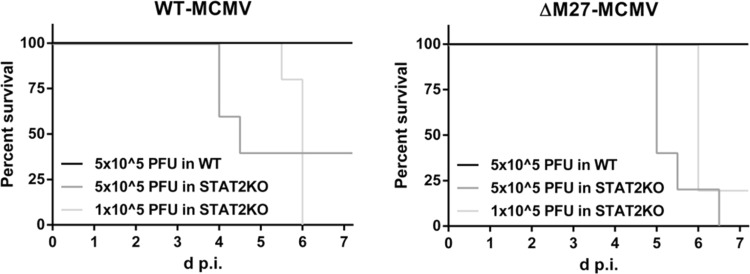
STAT2-deficient mice also succumb to ΔM27-MCMV infection. 129 and STAT2-knockout mice were infected i.p. with the indicated doses of wt-MCMV and ΔM27-MCMV. Infected animals were inspected twice a day. The number of moribund and dead mice was monitored to calculate the survival rates. The percentages of surviving animals (not moribund or dead) are depicted (*n* = 5).

The largely restored replication of ΔM27-MCMV in STAT2-deficient mice (Fig. 7) suggested that ΔM27-MCMV infection also leads to an increased severity of disease. To test this, we infected 129 control mice and STAT2-deficient mice with wt-MCMV and ΔM27-MCMV to monitor the disease progression. As shown in Fig. 8, STAT2-deficient mice were similarly susceptible to ΔM27-MCMV and wt-MCMV infection: within 1 week, most infected STAT2-deficient animals succumbed to infection irrespective of the presence or absence of pM27.

Taken together, our findings reveal a mutual influence in the evolutionary race between STAT2 and its antagonist, pM27, and uncover an evolutionary standoff situation with a delicate balance between host and virus.

## DISCUSSION

**FIG 9:**
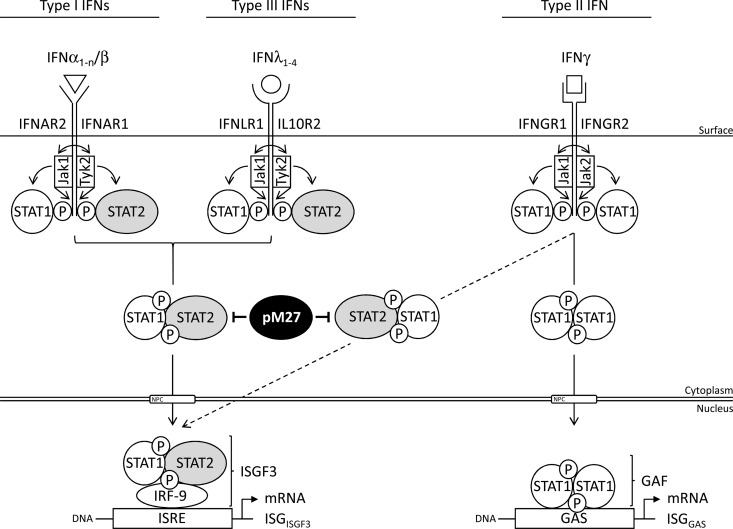
Role of STAT2 in IFN signaling. Schematic overview showing the signaling pathways of type I, II, and III interferons. In addition to the canonical role of the STAT1/2 proteins in all three pathways, the noncanonical role of STAT2 is depicted. IL10R2, interleukin-10 receptor 2; GAS, gamma-activated sequence; GAF, gamma-activated factor.

In this study, we analyzed the impact of the MCMV antagonist pM27 and its target, STAT2. Our data suggest a role of STAT2 for type I, II, and III IFN signaling (Fig. 9). We show that the pM27-mediated degradation of STAT2 impairs canonical type I as well as type III IFN signaling (Fig. 5). Type I and type III IFNs are all induced upon infection, albeit in a cell type-specific and temporally regulated manner (49). Thus, the lack of viral inhibition of IFN-λ responses in ΔM27-MCMV-infected mice might be an important factor contributing to the strong *in vivo* attenuation of the mutant. IFN-λ-induced interferon-stimulated gene (ISG) expression *in vivo* is evident in organs like the lungs and salivary glands (50), suggesting that MCMV is exposed to IFN-λ during its replication *in vivo*. The relevance of IFN-λ for CMV is further underlined by associations of IFN-λ single nucleotide polymorphisms with control of HCMV replication after transplantation (51).

Another interesting aspect of our study is the complete recovery of ΔM27-MCMV in STAT2-deficient cells upon IFN-γ treatment (Fig. 4). Although STAT1 phosphorylation and signaling were not profoundly disrupted (Fig. 4E), the severe IFN-γ susceptibility of ΔM27-MCMV was fully reverted. Our result joins previous findings which demonstrate that, in addition to the canonical STAT1-mediated IFN-γ signal transduction, STAT2 has a profound importance for the antiviral activity induced by IFN-γ (11, 12, 45, 46).

The antiviral activity of IFNs increases during the (pre)incubation time (52, 53). One noteworthy observation is that the STAT2 antagonist pM27 counteracts the antiviral activity elicited by IFNs even in experiments comprising IFN preincubation (Fig. 4). One might have hypothesized that IFN-induced effector proteins would have been translated prior to infection, establishing an antiviral state resistant to signaling antagonists. That this is not the case might be attributed to the feed-forward activation loop of IFNs: in addition to the upregulation of effector proteins, IFNs upregulate components of their own signaling pathway, like STAT1 and STAT2 (Fig. 4E). This enhanced signaling competence established during prolonged IFN exposure seems to be crucial for efficient antiviral activity. This interpretation is supported by the finding that inhibition of Janus kinase activity by ruxolitinib starting at the time point of infection completely reversed the antiviral activity elicited by IFN-γ against ΔM27-MCMV, despite a 2-day-long IFN preincubation period (Fig. 3B).

ΔM27-MCMV is severely attenuated *in vivo*. Replication was largely restored in all tested organs of STAT2-deficient animals (e.g., >1,000-fold recovery in spleen and liver at 5 days p.i.; Fig. 7). However, in contrast to the complete recovery of ΔM27-MCMV replication observed in IFN-conditioned cells *in vitro*, a small but statistically significant attenuation of ΔM27-MCMV in comparison to that of wt-MCMV remained in STAT2-deficient animals *in vivo* (Fig. 7). This indicates that pM27 possesses at least one additional function and/or target beyond STAT2 antagonism which is relevant *in vivo* but appears to be dispensable in fibroblasts *in vitro*.

On the basis of the findings that pM27-mediated STAT2 degradation affects type I, type II, and type III IFNs, the mutual influence can be analyzed only using ΔM27-MCMV and STAT2-deficient mice. Our data show that STAT2 and its downstream target genes are, on the one hand, pivotal determinants for the control of MCMV replication and, on the other hand, central targets of MCMV-mediated antagonism of the immune system. This highlights a standoff established by opposing evolution between host and pathogen which results in a situation in which the host achieves sufficient immune responses mediated via STAT2 to prevent disease and mortality. Simultaneously, MCMV evolved sufficient STAT2 antagonism to allow productive virus replication. If this delicate balance is shifted toward one direction, the stable equilibrium collapses, leading to fatal disease of the host or the inability of the virus to replicate. Our findings uncover that effective control of *in vivo* MCMV replication and the associated disease critically relies on STAT2, despite the existence of a potent MCMV-encoded STAT2 antagonist. This appears to be contradictory at first glance. In our opinion, three nonmutually exclusive explanations might contribute to the solution to this seeming paradox.

(i) The STAT2 degradation in MCMV-infected cells might not be efficient or fast enough to fully eliminate STAT2-dependent antiviral effects within infected cells. However, based on the fast and abundant pM27 expression, the kinetics and efficacy of STAT2 degradation, and the expression of additional MCMV-encoded inhibitors of IFN signaling (10), as well as IFN-induced effector functions (e.g., protein kinase R [54]), we anticipate that residual STAT2 amounts within infected cells have only a limited contribution to the STAT2-dependent control of wt-MCMV in productively infected cells, like fibroblasts, but may be more relevant in less permissive cells, like monocytes.

(ii) STAT2 in uninfected immune cells might impact MCMV control. Cytomegaloviruses can infect a variety of different cell types. However, as in the case of HCMV (55), viral gene or antigen expression occurs at best only sporadically (<2% of cells) in lymphocytes, like B and T cells, during the acute phase of viral replication (56, 57). Constitutive gene deletion, as in the case of STAT2-knockout mice, affects all cells, including lymphocytes, which are most likely not affected by pM27 in the wild-type mouse situation. Thus, STAT2 deficiency could impair the adaptive immune responses against MCMV. However, the increase in MCMV titers and the kinetics of disease progression in STAT2-deficient mice taking place in the first week after infection do not argue for an impairment of adaptive immunity. Jonjić and coworkers have shown that antibodies are not essential for the resolution of primary MCMV replication (58). In CD8-negative mice, “the kinetics of virus clearance was found to be almost indistinguishable from that of fully immunocompetent mice” (59). Similarly, “a selective deficiency of the CD4 subset does not prevent the control of CMV infection” (60). Severe combined immune-deficient (SCID) mice do not succumb before day 20 after infection with a standard dose of MCMV (e.g., see references 18 and 61), and NOD/SCID/IL-2rg^−/−^ mice, which additionally lack NK cells, die upon MCMV infection after day 12 (62). Collectively, we estimate the role of lymphocytes and adaptive immunity in the herein described STAT2-dependent phenotype during the first days of infection to be rather restricted at early time points. Consistently, cellular as well as humoral adaptive immune responses were largely normal in STAT2-deficient hamsters upon adenovirus type 5 infection (63).

(iii) A fraction of active STAT2 molecules might remain one step ahead of pM27's reach, irrespective of how fast pM27 acts, and behave like the tortoise in Zeno of Elea's paradox of Achilles and the tortoise. In contrast to pM27, IFNs are cytokines which act in an autocrine as well as a paracrine manner. Additionally, cell-intrinsic immunity produces cGAMP(2′-5′), which can also spread to bystander cells and induce STING-dependent IFN expression (64). Thus, IFN and cGAMP can condition neighboring cells, leading to STAT2 activation undisturbed by IFN antagonists like pM27. According to this model, cells harboring IFN-induced STAT2 activation surround the cells with current virus production and will be reached by virus and antagonist only one (or more) replication cycle later. Future work based on the results and conclusions of the herein described study will discriminate between these explanations and/or evaluate their respective contributions.

Irrespective of how STAT2 acts on the molecular level, its expression is essential for host survival even in the presence of a potent viral antagonist.

## MATERIALS AND METHODS

### Cells, cytokines, and compounds.

Primary mouse fibroblasts (mouse embryonic fibroblasts [MEF] and mouse newborn cells [MNC]) were isolated from C57BL/6 and BALB/c mouse embryos and newborns according to described protocols (65). Immortalized mouse fibroblasts were generated from primary C57BL/6 and BALB/c MEF by crisis immortalization (66). HeLa:IFNLR and 3T3-ISREluc:IFNLR cells were generated by transfection of HeLa cells (ATCC CCL-2) and NIH 3T3:ISREluc cells (11) with plasmids pcDNA-hIFNLR1 (subcloned from pUNO1-hIL28R; InvivoGen) and pUNO1-mIL28R (InvivoGen), respectively, and subsequent selection with the appropriate agent. All cells were grown in Dulbecco modified Eagle medium supplemented with 10% (vol/vol) fetal calf serum, 100 μg/ml streptomycin, 100 U/ml penicillin, and 2 mM glutamine. Cell culture media and supplements were obtained from Gibco/Life Technologies. Mouse IFN-α was purchased from PBL, mouse IFN-γ was purchased from Merck Millipore, human and mouse IFN-λ was purchased from R&D Systems, and ruxolitinib was purchased from Cell Guidance Systems.

### Viruses, infection conditions, and virus titration.

wt-MCMV and ΔM27-MCMV with mutated MCK-2 have been described previously (11). For the construction of ΔM27-MCMV with repaired MCK-2, the MCMV BAC described previously (28) was used. The virus reconstituted from this parental BAC served as the wt-MCMV control. The deletion of *M27* was done according to established protocols (67). Briefly, a PCR fragment was generated by use of the primers DeltaM27-Kana1 (GGAGCGAACAGAACCAGCCATATTCCTCCTGTCTTCCGTGTGTCGCTCGTGTCCTGTCA*CCAGTGAATTCGAGCTCGGTAC*; italic nucleotides indicate the part of the primers which bind to the template DNA; other nucleotides indicate the recombination site in the BAC sequence) and DeltaM27-Kana2 (GTGTGAGGAAAAAGACGACGGTGTCATTTATCGACGGCGCCGTGTCGCGCTGACCATG*GACCATGATTACGCCAAGCTCC*) and the plasmid pSLFRTKn (68) as the template. The PCR fragment containing a kanamycin resistance gene flanked by *frt* sites was inserted into the parental BAC by homologous recombination in Escherichia coli, leading to the replacement of the *M27* sequence by the kanamycin resistance cassette. This cassette was subsequently removed by FLP-mediated recombination. Correct mutagenesis was confirmed by Southern blotting and PCR analysis (data not shown). Recombinant MCMVs were reconstituted by transfection (Superfect; Qiagen) of BAC DNA into permissive fibroblasts. Viral titers were determined by standard plaque titration (69) on primary MEF or MNC (65). All *in vitro* infections and titrations were conducted with centrifugal enhancement (900 × *g* for 30 min). For *in vivo* infections, mice were infected intraperitoneally (i.p.) with MCMV. Organs of infected mice were harvested, snap-frozen in liquid nitrogen, and stored at −80°C until titrations were performed.

### Animal care.

C57BL/6 and BALB/c mice were obtained from Charles River or Harlan and housed in the animal facility of the Institute for Virology of the University Hospital Essen. 129 and STAT2-knockout mice were bred and housed in the same facility. STAT2-knockout mice were generated by the group of Christian Schindler (Columbia University, New York, NY, USA), as described in reference 42, and kindly shared with us by Stefan Jordan (at that time at the Ludwig Maximilians University, Munich, Germany).

### Ethics statement.

All procedures were done in accordance with European Union regulations and with the permission of the local authorities (the Landesamt für Natur, Umwelt, und Verbraucherschutz) in North Rhine Westphalia, Germany (permission number 84-02.04.2014.A390 for the MEF preparation and permission number 84-02.04.2013.A414 for MCMV *in vivo* infections).

### Luciferase assay.

Luciferase activity was measured according to the manufacturer's instructions (pjk) using a microplate luminometer (Mithras LB 943; Berthold).

### Immunoblot analysis.

Immunoblotting was performed according to standard procedures (see, e.g., see reference 53) using the following antibodies: anti-phospho-STAT1 (Cell Signaling Technology), anti-STAT1 (Santa Cruz), anti-STAT2 (Santa Cruz), anti-actin (Sigma), and anti-GAPDH (anti-glyceraldehyde-3-phosphate dehydrogenase; Santa Cruz). Proteins were visualized using peroxidase-coupled secondary antibodies and an enhanced chemiluminescence system (Cell Signaling Technology).
